# Prevertebral Soft-Tissue Swelling at C7 Is Highly Sensitive for Cervical Spine Ligamentous Injury Study Type: Retrospective Cohort Study

**DOI:** 10.5435/JAAOSGlobal-D-19-00093

**Published:** 2020-04-01

**Authors:** Jonathan C. Savakus, Douglas S. Weinberg, Timothy A. Moore, Heather A. Vallier

**Affiliations:** From the Department of Orthopaedic Surgery, MetroHealth Medical Center, Case Western Reserve University, Cleveland, OH.

## Abstract

To determine the diagnostic potential of prevertebral soft-tissue (PVST) swelling in cervical spine ligamentous injury (LI).

**Background:**

PVST swelling in the cervical spine is a historical indicator of cervical spine injury; however, at present, there are no limited objective criteria to use PVST swelling to guide clinical decision-making regarding cervical spine LI. This study investigates PVST thickness as a screening measure for cervical spine LI with a potential to identify indications for advanced imaging.

**Methods:**

The registry at an urban level 1 trauma center was queried for cervical spine injuries between 2010 and 2016. Twenty-nine patients with LIs who had both CT and MRI available were included. Fifty-nine patients with bony injury (BI) were also included, and 99 patients undergoing CT of the cervical spine after blunt trauma without evidence of cervical spine injury were included as control patients.

**Results:**

PVST swelling >11.5 mm at C7 was 89.7% sensitive (72.7% to 97.8%) and 51.5% specific (41.3% to 61.7%) for LI. In men, a PVST thickness of 11.5 mm at C7 was 96% sensitive (79.7% to 99.9%) and 46.2% specific (32.2% to 60.5%) for LI. Patients with LI were more likely to be men (86.2% versus 52.5% control, *P* < 0.01). 86.2% of patients with LI (25 of 29) had associated BI. Patients who had LI and no associated BI (n = 4) were all men, and all had PVST thickness >11.5 mm at C7 (avg. PVST 17.7 mm ± 2.5).

**Conclusion:**

C7 PVST thickness >11.5 mm was highly sensitive but poorly specific for cervical spine LI. This threshold may represent an appropriate PVST thickness to rule out LI because patients with PVST ≤11.5 mm are unlikely to have cervical spine LI and may not benefit from MRI.

The clinical criteria for identifying an uninjured cervical spine in patients who are awake and alert without distracting injury, neurologic deficit, intact range of motion, or cervicalgia is well established and highly sensitive.^1-3^ However, there remains the debate regarding patients who have altered mental status and for whom imaging must be solely relied on to clear the cervical spine.^3,4^ In these patients, CT is highly sensitive and specific for detecting bony injuries (BIs) and has largely replaced conventional radiography as the initial cervical spine imaging modality,^3^ although it is poorly sensitive in detecting ligamentous or vertebral disk injury. Although uncommon, injuries isolated to these structures can be unstable and can result in substantial neurologic deficit.^5^ The question of whether to supplement a negative CT scan with more advanced imaging, such as MRI, is currently a subject of debate. MRI is more costly than CT, and in an increasingly restricted payer environment, establishing the appropriate criteria for use remains especially relevant. Any failures to identify unstable cervical spine discoligamentous injuries (LIs) in setting of a negative CT scan may result in disastrous outcomes for the patient.

The most recent Eastern Association for Surgical Trauma guideline for cervical spine clearance in the unreliable patient conditionally recommends the removal of cervical collar after negative high-quality CT; a recommendation based on the strong negative predictive value of CT in excluding critically unstable injuries to the cervical spine, although the guidelines acknowledge that this recommendation is based only on low-quality evidence.^4^ In contrast to this recommendation, other authors have shown MRI to change the management in certain patients with negative admission CT by means of surgical intervention and/or continuation of cervical collar.^6-8^ Thus, it would be of value to determine which patients with negative admission CT would benefit from further evaluation with MRI. Ideally, a set of objective indicators of occult cervical spine injury on CT would be established. Some authors have already reported the retrospective finding of subtle CT signs of disco-LI after injuries were diagnosed acutely via MRI.^7-9^

Prevertebral soft-tissue (PVST) swelling has been classically used as an indicator of occult injury on cervical radiographs,^10,11,12,13,14,15,16,17^ with recent studies describing various normal values for PVST on CT imaging.^18,19^ On lateral cervical radiographs, a PVST thickness of greater than 6 mm at C2 or 22 mm at C6 was traditionally thought to indicate cervical spine injury. However, these values were shown to be more specific rather than sensitive for cervical spine injury and were not recommended as a screening tool because it is critical to establish a threshold that limits false negatives.^16^ A recent study examined the PVST thickness on CT and showed that the cutoff for injury of 1 to 2 SDs above the normal at C2 and C6 was a highly specific, yet poorly sensitive measure of occult cervical spine injury.^18^ No recent literature has examined the potential of lower thresholds of PVST thickness for injury; to the authors' knowledge, no study has ever examined PVST in the setting of cervical spine LI.

In the present study, we aim to examine the potential of PVST in all cervical vertebral levels and various PVST thicknesses as indicators of injury. It is our hypothesis that the measurement of PVST will prove to be a valuable and objective adjunct to the interpretation of a screening CT in cervical trauma and will help inform clinicians on which patients would or would not benefit from further imaging, such as MRI, for evaluation of cervical disco-LI.

## Methods

### Patients

After institutional review board approval, the electronic medical record at an urban level 1 trauma center was queried for cervical spine injuries between 2010 and 2016 to identify a cohort of individuals with traumatic cervical spine LIs. The presence of LI and the level of injury were determined based on final radiologist read of the MRI scan.^20^ A consecutive series of 32 patients who underwent MRI in addition to CT for cervical spine injury and were found to have LI were identified from the practice of a single, fellowship-trained spine surgeon. Patients were excluded if CT was performed at outside hospital (n = 3) where image quality and resolution could not be verified. Twenty-nine patients with LI remained and were included in the primary analysis.

To serve as a comparison, a second consecutive series of individuals with BI and no LI were established from the same clinical practice. BI was defined as a fracture on CT scan as documented in final radiologist read.^21^ To determine the appropriate number of patients with BIs to serve as a comparison to those with LIs, an a priori power analysis was conducted: assuming a significance of 0.05, power of 0.80, and effect size of 0.15 resulted in an estimated sample of 59 patients with BI. Thus, 86 patients over the same period were identified who underwent CT but not MRI for traumatic cervical spine injury. Patients who arrived intubated (n = 8), underwent initial CT at outside hospital (n = 12), had subacute presentations (n = 2), had nontraumatic mechanism of injury (n = 2), had previous spine instrumentation (n = 2), or had ankylosing spondylitis (n = 1) were excluded. In total, 59 patients with BIs and no LIs were included as a comparison group.

Finally, a control group of patients with no cervical spine injuries was established. An identical a priori power analysis was conducted, which revealed an estimated minimum sample size of 46 patients. Ninety-nine patients who underwent cervical spine CT but not MRI for traumatic indication were identified and were found, via a comprehensive clinical and radiographic assessment, to have no clinically evident injuries. These patients were included as uninjured control patients.

### CT Measurements

**Figure 1:**
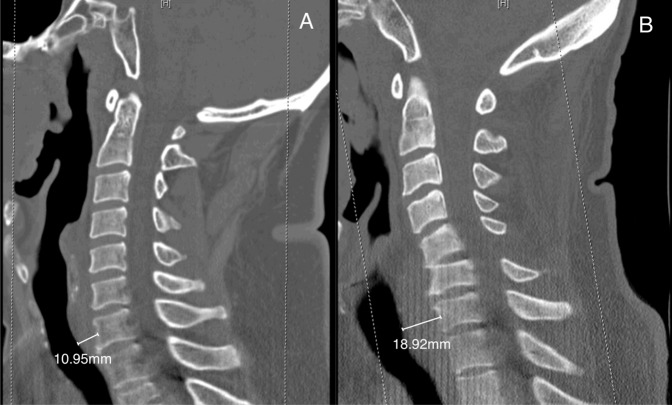
Radiographs showing the examples of measurements of prevertebral soft-tissue thickness for control patient (**A**) and patient with ligamentous injury (**B**). The midsagittal plane was determined via axial correlation. Measurements were taken from the midanterior of the vertebral body to the closest point in the air column in a plane approximately parallel to the vertebral end plate.

PVST thickness was measured in a previously described manner.^19^ Midsagittal planes were determined using direct axial image correlation. Measurements were taken from the midanterior portion of the vertebral body to the closest point in the air column in a plane approximately parallel to the vertebral end plates (Figure 1). Measurements were taken to the nearest 10th of a millimeter. Internal validity of the measurement technique was verified by calculating the intraobserver and interobserver variability in a random subset of patients at C2, C6, and C7. This was performed by two of the study authors on 20 patients.

### Statistical Analysis

All statistical analyses were performed with Minitab 17 (Minitab). Categorical variables were compared with the Fisher exact test, and continuous variables with Student *t*-test. Histograms were used to depict the distribution of various thickness of PVST as a function of density (proportion of patients in a group with corresponding PVST thickness) to correct for the smaller sample size of the ligamentous and BI groups. To generate the diagnostic value of PVST at each level, the likelihood ratios were generated to predict the amount of PVST for LI. Receiver operating curves were calculated via event probabilities for each patient's C7 PVST in relation to their injury status. Event probabilities were generated via binary logistic regression between C7 PVST thickness and the presence of LI. Significance was set at *P* ≤ 0.05.

## Results

### Patients

Twenty-nine patients with LIs with or without bony involvement and 59 patients with BIs and no confirmed LI were identified. Ninety-nine patients with no cervical spine injury were included as control patients. Patients with BI were significantly older (54.8 years versus 46.8; *P* = 0.03) than control patients. Patients with LI were more likely to be men than control patients (86.2% versus 52.5%, *P* < 0.01). No significant difference was observed in the body mass index between LI, BI, or control patients. 86.2% of patients with LI (25 of 29) also had associated BI. 93.1% of patients with LI underwent stabilization of their injuries, whereas 54.2% of patients with BI underwent surgical intervention (Table 1).

**Table 1:** Patient Groups and Injury Characteristics

	Ligament	Bone	Control Patients
N	29	59	100
Age (SD)	53 (19.2)	54.8 (21.9)^a^	46.8 (20.9)
Sex (% male)	86.2%^b^	54.2%	52.5%
BMI (SD)	26.7 (7)	27.7 (7.4)	28.7 (6.8)
Surgical management (%)	93.1	55.9	—
Multilevel injury (%)	75.9	66.1	—
Involvement of			
C1 (%)	6.9	15.3	—
C2 (%)	13.8	35.6	—
C3 (%)	13.8	5.1	—
C4 (%)	31.0	6.8	—
C5 (%)	58.6	33.9	—
C6 (%)	55.2	45.8	—
C7 (%)	31.0	35.6	—
Involvement of			
Anterior longitudinal ligament	65.5%	—	—
Posterior longitudinal ligament	44.8%	—	—
Posterior ligamentous complex	69.0%	—	—

BI = bony injury, BMI = body mass index, LI = ligamentous injury

a Indicates significant at alpha of 0.05 versus control.

b Indicates significance at alpha of 0.001 versus control.

Descriptive statistics of patients with LI (ligament), BI (bone), and no injury (control).

### Prevertebral Soft-Tissue Measurements

Measurements of PVST were taken at all cervical vertebral levels. The differences between the average PVST for each vertebral level were compared between the injury and control patient population (Table 2). The differences in PVST between sex were also calculated (Table 3). Statistically significant increases in PVST were seen in patients with BI at C2, C3, C6, and C7. Statistically significant increases in PVST thickness were seen in patients with LI at C3 and C5 to C7. Significant baseline differences were observed in PVST between sex in C1, C2, and C5. No significant baseline differences were observed in C7 PVST between men and women. Intraobserver variability was calculated to be 0.996 at C2, 0.953 at C6, and 0.922 at C7. Interobserver variability was calculated to be 0.921 at C2, 0.821 at C6, and 0.813 at C7. All reliabilities were considered to be “excellent.”^22^

**Table 2:** Average PVST Thickness in Injured Versus Control Patients

Vertebral Level	LI (n = 29)	BI (n = 59)	Control Patient (n = 99)
C1 (SD)	4.6 mm (3.2)	5 mm (3.1)	4.7 mm (2.2)
C2 (SD)	5.2 mm (3.2)	5.4 mm (2.7)^a^	4.3 mm (1.8)
C3 (SD)	6.5 mm (3.8)^b^	6.1 mm (3.2)^b^	4.9 mm (2.2)
C4 (SD)	10.6 mm (5)	9.4 mm (4)	9.2 mm (4.8)
C5 (SD)	16.6 mm (3.6)^a^	14.4 mm (3.7)	13.7 mm (3.6)
C6 (SD)	16.7 mm (4.2)^a^	14.8 mm (3.2)^b^	13.7 mm (2.7)
C7 (SD)	16 mm (4.7)^a^	14 mm (4.1)^a^	11.5 mm (3.1)

BI = bony injury, LI = ligamentous injury, PVST = prevertebral soft tissue

a Indicates significance at alpha of 0.001 versus control.

b Indicates significant at alpha of 0.05 versus control.

Average and SD of PVST thickness by injury group in all patients.

**Table 3:** Average PVST Thickness by Sex

Vertebral Level	Male Control Patient (n = 53)	Female Control Patient (n = 46)
C1 (SD)	5.6 mm (2.3)	3.6 mm (1.5)^a^
C2 (SD)	4.7 mm (1.8)	3.9 mm (1.8)^b^
C3 (SD)	5.2 mm (2.3)	4.6 mm (2)
C4 (SD)	9.4 mm (5.4)	8.9 mm (4)
C5 (SD)	14.5 mm (3.4)	12.8 mm (3.6)^b^
C6 (SD)	14.1 mm (2.5)	13.3 mm (2.9)
C7 (SD)	12 mm (2.9)	10.9 mm (3.3)

PVST = prevertebral soft tissue

a Indicates significance at alpha of 0.001 versus control.

b Indicates significant at alpha of 0.05 versus control.

Average and SD of PVST thickness by sex in control patients.

### Sensitivity and Specificity of Prevertebral Soft-tissue Thickness for Cervical Spine Injury

PVST swelling >11.5 mm at C7 was 89.7% sensitive (72.7% to 97.8%) and 51.5% specific (41.3% to 61.7%) for LI, with a positive likelihood ratio of 1.85 (1.46 to 2.35) and a negative likelihood ratio of 0.2 (0.07 to 0.60). In men only, a PVST thickness of 11.5 mm at C7 was 96% sensitive (79.7% to 99.9%) and 46.2% specific (32.2% to 60.5%) for LI, with a positive likelihood ratio of 1.82 (1.39 to 2.37) and a negative likelihood ratio of 0.08 (0.01 to 0.59). Patients who had LI and no associated BI (n = 4) were all men, and all had PVST thickness >11.5 mm at C7 (avg. PVST 17.7 ± 2.5). PVST thickness >17.5 mm at C7 was 31.0% sensitive (15.3% to 50.8%) and 96.0% specific (90% to 98.9%) for LI in all patients, with a positive likelihood ratio of 7.68 (2.55 to 23.14) and a negative likelihood radio of 0.72 (0.59 to 0.92). In men only patients, PVST thickness >17.5 mm was 32% sensitive (14.9% to 53.5%) and 96% specific (87% to 99.5%) for LI, with a positive likelihood ratio of 8.48 (1.94 to 37.06) and a negative likelihood ratio of 0.71 (0.54 to 0.93) (Table 4).

**Table 4:** Sensitivity and Specificity of C7 PVST in LI

	Sensitivity	Specificity
Male and female (n = 128)		
11.5 mm	89.6% (26/29)	52% (51/99)
17.5 mm	31% (9/29)	95.9% (95/99)
Male only (n = 78)		
11.5 mm	96% (24/25)	47% (25/53)
17.5 mm	32% (17/25)	96% (51/53)

BI = bony injury, LI = ligamentous injury, PVST = prevertebral soft tissue

Sensitivity and specificity of PSVT thickness in patients with LI compared with patients with no cervical spine injury. Patients with BI and no confirmed LI were not included in this analysis. Cutoff for injury was PVST thickness of 11.5 or 17.5 mm, respectively.

Positive and negative predictive values were calculated in conjunction with previously published incidence of any LI on MRI after negative CT of 16.2% patients.^8^ Positive and negative predictive values of a PVST cutoff of 11.5 mm were determined to be 29% and 96%, respectively. A PVST cutoff of 17.5 mm had positive and negative predictive values of 59% and 87%, respectively.

## Conclusion

The evaluation of PVST thickness as an indirect marker of cervical spine LI after blunt trauma is important and has the potential to expedite spine care and the trauma evaluation of these critically injured patients. In the present study, we found PVST thickness >11.5 mm at C7 to be highly sensitive but poorly specific for LI, with greatest sensitivity among male patients (Table 4). This equates to a negative predictive value of 96% for PVST <11.5 mm. The clinical utility of this finding should be interpreted primarily as a means of ruling out LI because patients with PVST <11.5 mm would be unlikely to have LI. This finding is in some contrast to conclusions drawn by previous studies of PVST thickness on CT imaging, which universally demonstrates poor sensitivity and high specificity for cervical spine injury.^17,18^ The differences between the present study and previous studies of PVST as a marker of injury can be condensed into differences between (1) the injuries studied, (2) the cutoff for injury, and (3) the vertebral level.

To the authors' knowledge, no previous studies of this subject have examined PVST thickness in LI. Because LIs are soft-tissue injuries, they may have greater propensity to present with increased soft-tissue swelling as compared to isolated BI. This is supported by the stepwise increase in mean PVST seen at C7 among control, BI, and LI patients (Table 2). Although it is possible that patients with BI and no confirmed LIs may have had undiagnosed LI, it is probable that they would have had a substantially lower burden of ligamentous involvement and therefore the increase in PVST swelling between BI and LI can be attributed to the additional soft-tissue involvement in the LI group. Importantly, the four patients found to have isolated LIs without associated BI had average PVST of 17.7 mm ± 2.5, which was statistically significantly higher than patients with LI and BI (*P* < 0.01). Among these patients, three had disk space abnormalities prompting MRI and one underwent MRI because of neurologic change. The latter patient had a stable injury and did not require surgery. Because isolated LI can still be unstable,^23^ it is important for these injuries to be identified promptly because they are more likely to be missed than other forms of spine trauma and can lead to catastrophic consequences. Thus, it is especially relevant to establish updated, appropriate PVST screening criteria using modern CT scan technologies.

**Figure 2:**
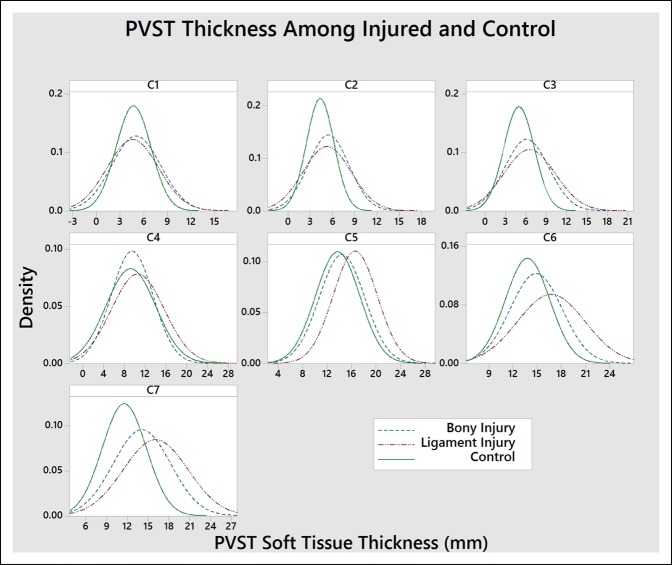
Histogram distribution showing PVST thickness at all vertebral levels in all patients. X-axis denotes PVST thickness in millimeters (mm). Y-axis density indicates the proportion of patients within each group with corresponding PVST thickness. PVST = prevertebral soft tissue

The traditional thresholds for lateral radiographs of the cervical spine of 6 mm at C2 and 22 mm at C6 are highly specific, and recent studies of PVST on CT using similarly large thresholds affirmed that these cutoffs of injury were very specific (Table 5). In the present study, we tested a wide range of thresholds to identify if an appropriate PVST threshold (cutoff) could be recommend with high sensitivity and lower specificity. This increased sensitivity would allow a large number of patients undergoing CT for traumatic indication to have LI ruled out after demonstrating PVST thickness below a certain threshold and thus be spared of an MRI for the purpose of evaluating for LI. We would argue that because the consequences of a missed diagnosis for unstable spine injury are of the utmost importance, it stands more value to establish a criterion that is especially sensitive for such. To our knowledge, the present study is one of the first attempts to do so. Furthermore, the choice of vertebral level is a critical difference in our study. The measurement at C7 demonstrated a greater separation between injured and control patients as compared with traditionally used C6 (Figure 2), allowing for more diagnostic discretion between injured and control patients. Although we would urge providers to focus on PVST at all levels, special importance should be paid at C7.

**Table 5:** Comparison With Previously Published Sensitivity and Specificity of PVST Thickness in Cervical Spine Injury at C2 and C6

	Sensitivity (95% CI)	Specificity (95% CI)
Present study		
C2	26.1% (17.3-36.6%)	89.9% (82.2-95.1%)
C6	32.9% (23.3-43.8)	83.8% (75.1-90.5%)
Previous study^18^		
C2	15.2% (10.5-21%)	89.4% (85.5-92.5%)
C6	22.1% (16.6-28.5%)	85.4% (81.1-89.5%)

CI = confidence interval, PVST = prevertebral soft tissue

Comparison of sensitivity and specificity of PVST thickness between the present data set and a recently published data set. Threshold for injury of 1 SD above control average PVST thickness was used. Both data sets are calculated with all injured patients (bony and ligamentous injury together) versus patients with no cervical spine injuries. Overlapping 95% CIs support the external validity of both studies.

The measurements reported in this study are similar to the literature standard. The internal validity of our study was consistent with excellent degrees of inter observer and intraobserver variability at C2, C6, and C7. The novel application of such at each level should give providers more confidence in interpreting their meaning. When comparing our control patients' mean PVST thickness to previously published normal values of PVST measured on CT, there was close agreement. A comparison with published normal values for PVST measured by CT demonstrated agreement within one SD at C2, C3, and C6, although these levels were statistically significantly different between studies. Alternatively, no statistically significant difference was observed at C7 (11.5 ± 3.1 mm versus 11.6 ± 3.2 mm).^19^ This result is encouraging because consistency in baseline PVST measurement at C7 supports its potential utility as a diagnostic tool. Although PVST at C7 has important diagnostic utility, PVST at other levels was less useful. We suspect this is because of a multitude of factors. With increased PVST at baseline, small changes will be easier to identify (larger delta). As a more cephalad location, gravity may bring more fluid in this direction. This is also a more common location for injury.^5^

**Figure 3:**
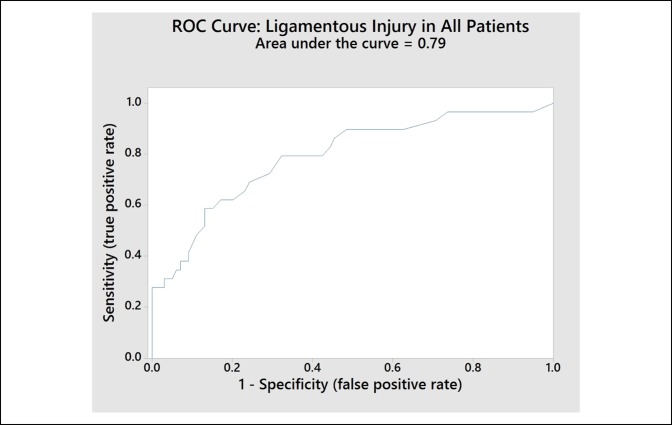
Graph showing receiver operating curve for PVST thickness at C7 in LIs. Area under the curve of 0.79 indicates acceptable discrimination between LIs and control patients on the basis of C7 PVST alone. LI = ligamentous injury, PVST = prevertebral soft tissue

Furthermore, we explored the accuracy of our sensitivity analysis by partially replicating the statistical methods of the most recent study of the diagnostic potential of PVST measured via CT (Figure 3). Using this previously published cutoff for injury of one SD above the control average, all 95% confidence intervals for sensitivity and specificity of PVST thickness at C2 and C6 overlapped between studies and were poorly sensitive and highly specific.^18^ This agreement supports the external validity of our study methodology and highlights the importance of PVST cutoff for injury and specific vertebral level measured (Table 5).

There were important limitations. At our center, MRI is not routinely ordered on patients with obvious LI evident on CT scan. Therefore, the actual number of patients with LIs was likely higher than reported because there were certainly individuals who had LI but did not meet the inclusions criteria based on the lack of MRI. However, to our knowledge, this study still represents the largest collection of patients with LI confirmed on both CT and MRI scan compared with a BI comparison group and noninjury control patient group. An additional limitation was the overwhelmingly high proportion of men in our LIs group. This is likely because of higher energy mechanisms of injury because men have been previously shown to be more likely the victims of extreme trauma.^24^ When removing all female patients from analysis, however, sensitivity increased from 89.6% to 96%, whereas specificity decreased from 52% to 47%. Similarly, a small difference was appreciated between BI and control patients regarding age. This is likely because of the inverse relationship between age and bone density, with older individuals having greater propensity to have a fracture after trauma. No difference in age was observed between LI and control patients, and all groups were similar in body mass index. The time point from injury to CT scan was not recorded, and it is possible that certain LIs may have evolved over time. In addition, all of the patients in this study were from a single surgeon's clinical practice.

The LIs included in this study also represented a population with most unstable injuries treated surgically. Therefore, the data on PVST thickness seen in the present work may not be reflective of all LI, rather those unstable LIs requiring fixation. It is well established that some patients with negative initial CT will have additional discoligamentous abnormalities detected on MRI, with recent estimates of these injuries ranging from 3.5% to 23.6%.^7,8,25^ Given the clinically relevant difference in incidence between stable and unstable LIs, one interpretation of these data are that PVST thickness is highly sensitive for *unstable* cervical LI. It is also possible that data on PVST thickness would be less prominent in stable LIs, secondary to less disruption of the native tissue architecture, although further research will be required to support this conclusion.

Practically speaking, when reviewing PVST in our own practice, we look for symmetry, asymmetry, and the presence of air and soft-tissue swelling in addition to any bony abnormalities. Although the results of this study indeed identify a statistically important threshold, we would still encourage providers to rely on all available clinical information and only as an adjunct to help aid in diagnosis.

The utility and clinical applicability of this study should focus primarily on PVST thickness as a mean to rule out cervical spine LI. Although CT scan can occasionally identify an injury, we would remind providers the true utility of this test is in ruling out injury because LI is most definitively diagnosed by MRI. PVST thickness of <11.5 mm at C7 had excellent negative predictive value (96%) and could theoretically eliminate the need for MRI for the purposes of evaluating LI in one half of the patients with negative CT. For patients with C7 PVST from 11.5 to 17.5 mm, our study data were not capable of differentiating injured from control patients and clinicians should rely on existing protocols for clearing the cervical spine. Finally, C7 PVST of >17.5 mm had high specificity (96%) for LI, but only moderate positive predictive value for having a LI (59%), given the relatively low incidence of LIs.
